# SOX9 gene shows association with adolescent idiopathic scoliosis predisposition in Northwest Indians

**DOI:** 10.1186/s40001-024-01635-8

**Published:** 2024-01-20

**Authors:** Hemender Singh, Shipra, Manish Gupta, Nital Gupta, Geetanjali Gupta, Ajay K. Pandita, Rajesh Sharma, Sarla Pandita, Vinod Singh, Bhavuk Garg, Ekta Rai, Swarkar Sharma

**Affiliations:** 1 Human Genetics Research Group, School of Biotechnology, Shri Mata Vaishno Devi University, Katra, India; 2 Department of Orthopaedics, All India Institute of Medical Sciences, New Delhi, India; 3 District Hospital Poonch, Poonch, Jammu and Kashmir India; 4 Department of Radiology, Shri Mata Vaishno Devi Narayana Superspeciality Hospital, Katra, Jammu and Kashmir India; 5 Accidental Hospital, Chowki Choura, Jammu, Jammu and Kashmir India; 6 Government Medical College, Jammu, Jammu and Kashmir India; 7 Chest Disease Hospital, Bakshi Nagar, Jammu, Jammu and Kashmir India; 8 Department of Orthopaedics, All India Institute of Medical Sciences, New Delhi, India; 9 School of Life Sciences, Jawaharlal Nehru University, New Delhi, India; 10 Human Genetics Research Lab, Centre for Molecular Biology, Central University of Jammu, Jammu, India

**Keywords:** Scoliosis, Genetic heterogeneity, *SOX9*, Case–control, High-throughput genotyping

## Abstract

**Background:**

Adolescent idiopathic scoliosis (AIS) is a common structural deformity of the spine affecting adolescent individuals globally. The disorder is polygenic and is accompanied by the association of various genetic loci. Genetic studies in Chinese and Japanese populations have shown the association of genetic variants of *SOX9* with AIS curve severity. However, no genetic study evaluating the association of SRY-Box Transcription Factor 9 (*SOX9*) variants with AIS predisposition has been conducted in any Indian population. Thus, we aimed to investigate the association of the genetic variants of the *SOX9* along with 0.88 Mb upstream region with AIS susceptibility in the population of Northwest India.

**Methods:**

In total, 113 AIS cases and 500 non-AIS controls were recruited from the population of Northwest India in the study and screened for 155 genetic variants across the SOX9 gene and 0.88 Mb upstream region of the gene using Global Screening Array-24 v3.0 chip (Illumina). The statistical significance of the Bonferroni threshold was set at 0.000322.

**Result:**

The results showed the association of 11 newly identified variants; rs9302936, rs7210997, rs77736349, rs12940821, rs9302937, rs77447012, rs8071904, rs74898711, rs9900249, rs2430514, and rs1042667 with the AIS susceptibility in the studied population. Only one variant, rs2430514, was inversely associated with AIS in the population, while the ten variants were associated with the AIS risk. Moreover, 47 variants clustered in the gene desert region of the SOX9 gene were associated at a *p*-value ≤ 0.05.

**Conclusion:**

The present study is the first to demonstrate the association of SOX9 enhancer locus variants with AIS in any South Asian Indian population. The results are interesting as rs1042667, a 3' untranslated region (UTR) variant in the exon 3 and upstream variants of the SOX9 gene, were associated with AIS susceptibility in the Northwest Indian population. This provides evidence that the variants in the enhancer region of *SOX9* might regulate its gene expression, thus leading to AIS pathology and might act as an important gene for AIS susceptibility.

**Supplementary Information:**

The online version contains supplementary material available at 10.1186/s40001-024-01635-8.

## Background

Numerous genetic loci have been identified to be associated with the adolescent idiopathic scoliosis (AIS) predisposition [1–8]. Several genetic studies have highlighted the importance of *SOX9* in the pathogenesis of AIS [3, 9]. The SOX9 gene is a master transcription factor for chondrogenesis [10]; mutations in the gene were found to be associated with many disorders, including kyphoscoliosis, sex reversal, bending of long bones, and others [11]. The altered expression of the SOX9 gene in the mesenchymal stem cells of AIS patients has highlighted its role in the AIS pathogenesis [9]. It has been found that *SOX9* is required for spine homeostasis in mice and drives the chondrogenesis in the spinal growth plate [12].

Moreover, a genetic study revealed that a genetic variant rs12946942, located near the SOX9 gene, was found to be associated with the severity of the AIS curve in Japanese and Chinese populations [4]. It has been established that the regulatory domain surrounding the SOX9 gene spans more than 2 Mb, consisting of multiple enhancers that alter the expression of the SOX9 gene during development [13], more specifically, a region located between 91 to 974 kb upstream of the *SOX9* has shown enhancer marks [14]. Based on the rationale of the importance of *SOX9* and its upstream region in the genetic predisposition of skeletal disorders and AIS, the screening of genetic variants across *SOX9* and 0.88 Mb upstream region was performed using high-throughput genotyping. The present study primarily aims to evaluate the association of the SOX9 gene with AIS susceptibility in the Northwest Indian population in a case–control study design.

## Materials and methods

### Study design

The present study is a case–control population-based genetic epidemiology study**.**

### Sample collection

Samples for the present study were collected from the school-based screening programme (the detailed characteristics of the population have been represented in our previous study) [15], followed by strict clinical evaluations after seeking informed consent from the individuals. The general characteristics of the cases and controls are provided in Table 1. Additionally, clinically diagnosed samples were recruited in the study from the All India Institute of Medical Sciences, New Delhi. All the cases were clinically diagnosed based on the Cobb angle measurement [16, 17]. The inclusion criteria for the cases were based on a Cobb angle measurement of 10° or greater. The cases and control samples recruited in the present study lie in the age group of 10–28 years. While cases were selected based on the age of onset of scoliosis, between 10 and 18 years. The control samples recruited in the present study were 21 years or older till 28 years identified during the population screening (the control selected in the present study were intentionally taken over 21 years to rule out any false negative case of late age onset of AIS, that would have remained undetected during adolescent but might have diagnosed few years later). The 2 millilitres of blood samples were collected from both the cases and controls in EDTA vials and stored at − 20 °C till further processing. A total sample size of 613 participants, comprising 113 cases and 500 non-AIS controls of Northwest Indian ethnicity (including various caste groups: Rajputs, Baniya, Brahmins, Kashmiri Pandits, Kashmiri Muslims, Sikhs, Buddhists, and many more) were recruited in the study. Of 113 AIS cases, 53 were males, and 60 were female.

**Table 1:** General characteristics of AIS cases and controls of the Northwest Indian population

	Average height in metres (± SD)	Average weight in kg (± SD)	Average BMI in kg/m^2^ (± SD)
Cases	1.63(± 0.10)	51.86 (± 12.43)	19.56 (± 4.43)
Controls	1.59(± 0.20)	50.63 (± 12.38)	19.86 (± 4.36)

*SD* standard deviation, *BMI* body mass index

### DNA isolation and genotyping

The genomic DNA of each participant was extracted from the peripheral blood sample utilising the XpressDNA Blood Kit (MagGenome^®^). The DNA was further processed for qualitative and quantitative analysis through the utilisation of Nanodrop 2000/2000c (ThermoFisher, USA) and Agarose gel electrophoresis. All the samples were genotyped using an Infinium Global Screening Array (GSA)-24 v3.0 chip (Illumina).

### Statistical analysis

The genotyping data were analysed using PLINK v1.07 and v1.09 [18]. A comprehensive analysis of the SOX9 gene and its 0.88 Mb upstream region revealed a total of 155 variations. The variants were obtained through the genotyping data using the PLINK flag “–extract” and subsequently subjected to filtration based on genotype and minor allele frequency, with thresholds set at “–mind 0.05” and “–maf 0.05”, respectively, as well as a genotyping rate threshold of “–geno 0.1”. Furthermore, the study utilised a predetermined threshold of 0.0005 to assess the Hardy–Weinberg equilibrium (HWE). Ten cases and five controls were excluded from the study due to a low call rate of less than 95% or being identified as ethnic outliers. This determination was made through principal component analysis after merging data from the UK Biobank and 1000 Genomes Phase 3 with our population data. The variants that passed the quality control assessment were subjected to an association test utilising the parameters –assoc –ci 0.95. The statistical significance of the association with AIS was evaluated at a Bonferroni correction of *p*-value = 0.000322. The regional association plot of filtered variants was generated using the LDassoc tool [19] using South Asians (SAS) as the reference population. The linkage disequilibrium (LD) of all variants was assessed using the Haploview tool [20]. The associated variants were further annotated using the variant effect predictor (VEP) tool from Ensembl [21, 22] and the UCSC genome browser [23, 24].

## Results

The Cobb angle in the cases ranges from 18.9° to 101° bending of the spine. The average Cobb angle of the AIS cases recruited in the study was 57.3°, with thoracic curvature being the prevalent type.

In the present study, out of 155 variants, 135 variants passed the quality test after filtering the genotyping data (HWE in controls ≥ 0.0005 and minor allele frequency in controls ≥ 0.05). From 135 variants, 11 variants were found to be significantly associated with the AIS genetic predisposition in the population of Northwest India (Table 2). Moreover, 47 variants were able to cross the p-value threshold of ≤ 0.05 in the present study, indicating that the association of the variants of the SOX9 gene does not appear by chance in the studied population group. All the variants other than the 11 significantly associated variants are tabulated in Additional file 2: Table S1.

**Table 2:** List of variants significantly associated with the AIS susceptibility in the Northwest Indian population at Bonferroni p-value threshold of 0.000322

Chr	Variant	MA	MAF_A	MAF_U	P	OR (95%CI)
17	rs9302936	A	0.2345	0.127	3.90 ˟ 10^–5^	2.105 (1.468–3.019)
17	rs7210997	A	0.2568	0.1552	0.000308	1.881 (1.329–2.661)
17	rs77736349	A	0.125	0.05499	0.000173	2.455 (1.516–3.974)
17	rs12940821	A	0.2227	0.1268	0.000254	1.973 (1.364–2.855)
17	rs9302937	G	0.1759	0.09165	0.000283	2.116 (1.402–3.194)
17	rs77447012	G	0.1343	0.05769	7.55 ˟ 10^–5^	2.533 (1.577–4.068)
17	rs8071904	A	0.1171	0.05172	0.000314	2.432 (1.48–3.997)
17	rs74898711	A	0.1441	0.05285	1.39 ˟ 10^–6^	3.019 (1.892–4.816)
17	rs9900249	A	0.1712	0.07825	2.06 ˟ 10^–5^	2.433 (1.599–3.701)
17	rs2430514	C	0.3198	0.4554	0.000229	0.5624 (0.413–0.766)
17	rs1042667	C	0.4037	0.276	0.000195	1.776 (1.31–2.408)

*Chr* chromosome, *MA* minor allele, *MAF_A* minor allele frequency in cases, *MAF_U* minor allele frequency in controls, *P* P-value, *OR* odds ratio, *CI* confidence interval

**Fig. 1:**
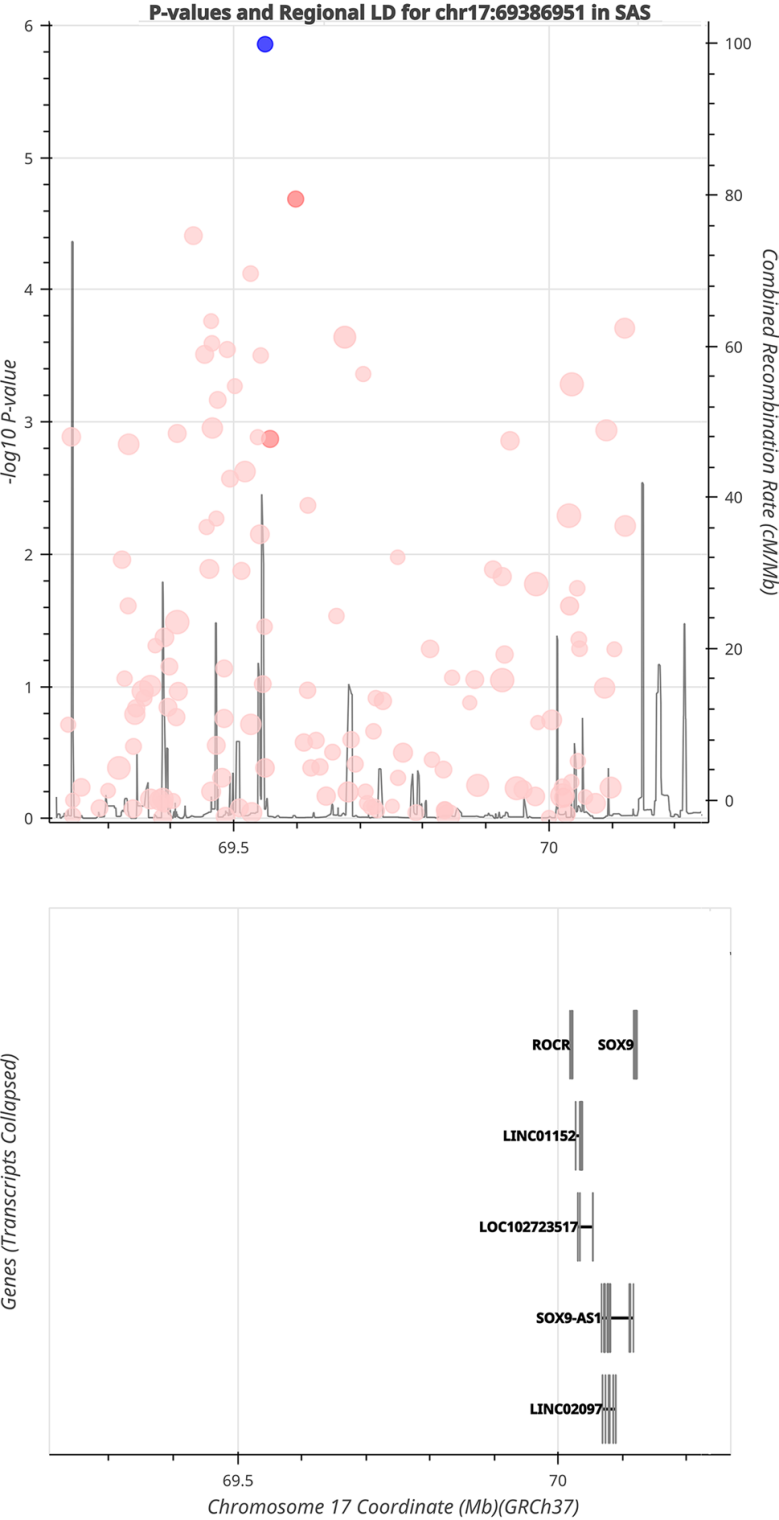
Regional association plot representing 135 variants that passed the quality test in the *SOX9* and 0.88 Mb upstream of the gene

The associated variants were further annotated using the Ensembl VEP tool and the UCSC genome browser. It was observed that variant rs1042667 was present at the 3' UTR region in exon 3 of the SOX9 gene; thus, it might play a role in the gene expression of the SOX9 gene. In contrast, all other associated variants were intergenic (Additional file 2: Table S1). The regional association of 135 variants that passed after filtering the genotyping data is represented in Fig. 1. Furthermore, the linkage disequilibrium plot of all 155 variants of *SOX9* was generated using the r-squared values. The dark black colour of the boxes represents variants in high LD in the studied population (Additional file 1: Figure S1).

## Discussion

Studies have extensively investigated the functional role of the SOX9 gene. 517–595 Kb gene desert upstream of the SOX9 gene promoter is related to the regulation of the gene in gonads [25]. The role of variants in and near the SOX9 gene in the pathogenesis of various skeletal disorders has been noted. The reciprocal translocation in the breakpoint position located 203 kb upstream of the SOX9 gene was found to be associated with acampomelic campomelic dysplasia [26]. Similarly, microdeletion on either side of the *SOX9* was associated with Pierre Robin sequence malformation [27, 28]. Genetic variations mapped to 1.3 Mb upstream and downstream of *SOX9* have been associated with several skeletal abnormalities and other disorders [29]. Numerous studies have indicated the importance of *SOX9* expression in the chondrocyte and bone development [10, 30–33].

In the present study, screening of genetic variants in the upstream region and across the SOX9 gene was performed in the population of Northwest India. Interestingly, out of 135 variants, 11 newly identified variants (Table 2) were associated with AIS genetic predisposition in the studied population group. All the variants except rs1042667 were observed to be intergenic/intronic in nature. The annotation of the variants using VEP and UCSC showed no significant functional consequences. One of the associated variants rs1042667 [OR 1.776 (1.31–2.408, 95% CI), p-value 0.000195] that passed the Bonferroni correction threshold is from the 3' UTR region of SOX9 gene, and we believe the variant might have a regulatory role on the SOX9 gene expression and thus the association.

Interestingly, the earlier reported intergenic variant, rs12946942 in the 17q24.3 locus, upstream of the SOX9 gene, did not show an association in the present study. This variant has shown an association with the AIS curve severity in the Japanese [4] and Han Chinese [4] populations and was replicated in an international meta-analysis with AIS severity [34]. However, a study in Chinese ancestry has also shown a lack of association of the variant rs12946942 with AIS curve severity [35].

We hypothesised that the differences are potentially due to genetic heterogeneity and differences in LD structure in the region. We further evaluated the LD of rs12946942 with the associated variants that were found in the present study. As anticipated, the variants did not show strong LD with the previously reported variant rs12946942 (~ D´ = 0.5, and ~ R^2^ = 0.02) in the SAS population. This substantiates our hypothesis of the possibility of the LD structure differences and evidence of genetic heterogeneity in different populations for AIS predisposition.

## Limitations of the study

This is the first genetic replication study of AIS susceptibility in any South Asian Indian (SAI) population group. The study still includes a small size of the population. It is pertinent to focus on the large sample size-based replication studies in the Indian population to replicate the association results.

## Strength of the study

No dataset from the region is available for comparisons of the association of the *SOX9* genetic variants. The present study is baseline data for *SOX9* genetic variants association with AIS in the Indian population. Also, even though the incidence of AIS is low, the present study will be a reference for future studies of AIS in SAI populations.

## Conclusion

The study hints towards the genetic heterogeneity in the Asian population. More replication studies are warranted in other population groups to identify the role of this region in AIS susceptibility to the respective population group and understand genetic heterogeneity in a better way. Also, functional studies are needed to validate the region, as an enhancer of the SOX9 gene, related to scoliosis and associated phenotypes.

### Supplementary Information

**Additional file 1: Figure S1. **Linkage disequilibrium plot of all the genetic variants screened across the locus, including the SOX9 gene in the Northwest Indian population.

**Additional file 2: Table S1. **List of variants studied in the Northwest Indian population in addition to variations that passed the Bonferroni Correction Threshold.

## Data Availability

All the data related to the study have been provided in the manuscript.

## Abbreviations

AIS: Adolescent idiopathic scoliosis
SOX9: SRY-Box Transcription Factor 9
UTR: Untranslated region
HWE: Hardy–Weinberg equilibrium
SAS: South Asians
LD: Linkage disequilibrium
VEP: Variant effect predictor
