# Supplementary material for: SOX9 gene shows association with adolescent idiopathic scoliosis predisposition in Northwest Indians
Source: Eur J Med Res. 2024 Jan 20;29:66. doi: 10.1186/s40001-024-01635-8 (PMC10799485; doi:10.1186/s40001-024-01635-8)
Supplement: Supplementary file 2 — Additional file 2: Table S1. List of variants studied in the Northwest Indian population in addition to variations that passed the Bonferroni Correction Threshold. [file 40001_2024_1635_MOESM2_ESM.docx]

**Additional file**

**Figure S1:** Linkage disequilibrium plot of all the genetic variants screened across the locus, including the SOX9 gene in the Northwest Indian population

**Table S1:** List of variants studied in the Northwest Indian population in addition to variations that passed the Bonferroni Correction Threshold.

| **S.No.** | **CHR** | **SNP** | **A1** | **F_A** | **F_U** | **P** | **OR (95% CI)** |
| --- | --- | --- | --- | --- | --- | --- | --- |
|  | 17 | rs12946942 | A | 0.1396 | 0.109 | 0.195 | 1.327 (0.864-2.039) |
|  | 17 | rs9909596 | A | 0.2928 | 0.1949 | 0.001296 | 1.71 (1.23-2.378) |
|  | 17 | rs2886917 | G | 0.06944 | 0.06301 | 0.7267 | 1.11 (0.6187-1.991) |
|  | 17 | rs7217041 | G | 0.0991 | 0.1004 | 0.9532 | 0.9855 (0.6054-1.604) |
|  | 17 | rs2158906 | G | 0.1964 | 0.2131 | 0.5803 | 0.9026 (0.6275-1.298) |
|  | 17 | rs11077554 | A | 0.1835 | 0.1775 | 0.8342 | 1.041 (0.7123-1.523) |
|  | 17 | rs78925994 | A | 0.06481 | 0.05601 | 0.615 | 1.168 (0.6372-2.142) |
|  | 17 | rs2058007 | G | 0.3874 | 0.3582 | 0.4137 | 1.133 (0.8395-1.53) |
|  | 17 | rs12947455 | A | 0.25 | 0.1762 | 0.01101 | 1.559 (1.105-2.199) |
|  | 17 | rs969264 | G | 0.1027 | 0.06925 | 0.08737 | 1.538 (0.9358-2.528) |
|  | 17 | rs78304940 | A | 0.1713 | 0.1154 | 0.0246 | 1.585 (1.058-2.374) |
|  | 17 | rs9908514 | C | 0.2661 | 0.3804 | 0.001475 | 0.5904 (0.4257-0.8189) |
|  | 17 | rs4019227 | C | 0.1574 | 0.1629 | 0.8433 | 0.9601 (0.6411-1.438) |
|  | 17 | rs77605527 | G | 0.1455 | 0.1191 | 0.2845 | 1.258 (0.8255-1.918) |
|  | 17 | rs2367385 | A | 0.3045 | 0.3541 | 0.1625 | 0.7988 (0.5827-1.095) |
|  | 17 | rs17766172 | A | 0.2611 | 0.2163 | 0.1464 | 1.28 (0.9169-1.787) |
|  | 17 | rs2190708 | C | 0.3796 | 0.3228 | 0.1087 | 1.284 (0.9457-1.743) |
|  | 17 | rs7212838 | A | 0.2182 | 0.1738 | 0.1227 | 1.327 (0.9257-1.902) |
|  | 17 | rs7221046 | C | 0.3604 | 0.4206 | 0.09943 | 0.7762 (0.5739-1.05) |
|  | 17 | rs2367391 | A | 0.3288 | 0.3419 | 0.7107 | 0.9431 (0.6919-1.285) |
|  | 17 | rs117751948 | A | 0.08559 | 0.05152 | 0.04922 | 1.723 (0.996-2.981) |
|  | 17 | rs76101219 | A | 0.4273 | 0.4143 | 0.724 | 1.055 (0.7846-1.418) |
|  | 17 | rs10512568 | G | 0.2162 | 0.2159 | 0.9914 | 1.002 (0.7033-1.427) |
|  | 17 | rs9302934 | G | 0.3636 | 0.3773 | 0.705 | 0.943 (0.6959-1.278) |
|  | 17 | rs7220313 | G | 0.2098 | 0.201 | 0.7675 | 1.055 (0.7381-1.509) |
|  | 17 | rs726099 | G | 0.2315 | 0.1725 | 0.04257 | 1.445 (1.011-2.065) |
|  | 17 | rs917345 | G | 0.1757 | 0.1375 | 0.1438 | 1.337 (0.9048-1.976) |
|  | 17 | rs199671746 | C | 0.177 | 0.1308 | 0.07079 | 1.429 (0.9687-2.107) |
|  | 17 | rs12325676 | G | 0.08716 | 0.09451 | 0.7353 | 0.9147 (0.5456-1.534) |
|  | 17 | rs6501483 | G | 0.1982 | 0.2413 | 0.1708 | 0.7773 (0.5417-1.115) |
|  | 17 | rs9891936 | A | 0.5505 | 0.4705 | 0.03264 | 1.378 (1.026-1.85) |
|  | 17 | rs9891945 | A | 0.2176 | 0.1316 | 0.001222 | 1.836 (1.265-2.663) |
|  | 17 | rs12948076 | G | 0.1757 | 0.1341 | 0.1091 | 1.376 (0.9301-2.034) |
|  | 17 | rs57602904 | A | 0.156 | 0.09324 | 0.006238 | 1.797 (1.175-2.748) |
|  | 17 | rs6501489 | A | 0.25 | 0.1772 | 0.01293 | 1.548 (1.095-2.189) |
|  | 17 | rs1034851 | G | 0.2117 | 0.1972 | 0.6242 | 1.094 (0.7644-1.565) |
|  | 17 | rs2367537 | G | 0.2703 | 0.387 | 0.001111 | 0.5867 (0.4249-0.8103) |
|  | 17 | rs34187161 | G | 0.2342 | 0.2016 | 0.2794 | 1.211 (0.8556-1.714) |
|  | 17 | rs73344709 | A | 0.125 | 0.06869 | 0.005376 | 1.937 (1.208-3.107) |
|  | 17 | rs12450307 | G | 0.1892 | 0.1066 | 0.0006813 | 1.956 (1.321-2.897) |
|  | 17 | rs35446272^#^ | A | 0.1045 | 0.02935 | 6.71E-07 | 3.861 (2.187-6.816) |
|  | 17 | rs7217976 | C | 0.208 | 0.2292 | 0.4905 | 0.883 (0.6198-1.258) |
|  | 17 | rs7216083 | A | 0.2566 | 0.215 | 0.1749 | 1.26 (0.9017-1.762) |
|  | 17 | rs17179607 | G | 0.1306 | 0.1808 | 0.07312 | 0.6808 (0.4462-1.039) |
|  | 17 | rs1014217 | A | 0.2431 | 0.2444 | 0.9677 | 0.993 (0.7055-1.398) |
|  | 17 | rs12945062 | A | 0.2455 | 0.1602 | 0.002684 | 1.705 (1.2-2.423) |
|  | 17 | rs73344733^#^ | A | 0.1308 | 0.04478 | 1.98E-06 | 3.211 (1.941-5.314) |
|  | 17 | rs16976703 | A | 0.1339 | 0.06508 | 0.000536 | 2.221 (1.4-3.524) |
|  | 17 | rs16976707^#^ | G | 0.03571 | 0.04049 | 0.7409 | 0.8778 (0.4051-1.902) |
|  | 17 | rs34980332^#^ | G | 0.01786 | 0.01521 | 0.7738 | 1.177 (0.3869-3.581) |
|  | 17 | rs17766911 | A | 0.2832 | 0.2904 | 0.8295 | 0.9654 (0.7006-1.33) |
|  | 17 | rs10512572 | A | 0.2064 | 0.14 | 0.01338 | 1.598 (1.1-2.324) |
|  | 17 | rs77388116^#^ | A | 0.1116 | 0.04453 | 9.17E-05 | 2.695 (1.612-4.507) |
|  | 17 | rs7224905 | G | 0.3636 | 0.2618 | 0.002372 | 1.612 (1.183-2.196) |
|  | 17 | rs76396838^#^ | A | 0.08257 | 0.04776 | 0.0398 | 1.794 (1.02-3.155) |
|  | 17 | rs12325887 | A | 0.3955 | 0.349 | 0.1937 | 1.22 (0.9036-1.648) |
|  | 17 | rs7207217 | A | 0.3009 | 0.3052 | 0.9028 | 0.9802 (0.7108-1.352) |
|  | 17 | rs12452395 | A | 0.1216 | 0.06008 | 0.001303 | 2.166 (1.339-3.504) |
|  | 17 | rs76516290^#^ | A | 0.06422 | 0.03138 | 0.02057 | 2.119 (1.107-4.054) |
|  | 17 | rs11077571 | A | 0.3716 | 0.2794 | 0.007076 | 1.525 (1.12-2.076) |
|  | 17 | rs35155380 | G | 0.211 | 0.164 | 0.0961 | 1.364 (0.9454-1.967) |
|  | 17 | rs13380873^#^ | A | 0.09009 | 0.03666 | 0.0006401 | 2.602 (1.475-4.588) |
|  | 17 | rs12172839 | A | 0.1622 | 0.1112 | 0.03537 | 1.547 (1.028-2.327) |
|  | 17 | rs113620309^#^ | A | 0.1171 | 0.02429 | 3.40E-10 | 5.328 (2.996-9.475) |
|  | 17 | rs75343260 | C | 0.211 | 0.2368 | 0.4136 | 0.8618 (0.6031-1.231) |
|  | 17 | rs72848742^#^ | G | 0.03125 | 0.02227 | 0.427 | 1.416 (0.5975-3.358) |
|  | 17 | rs4793364 | A | 0.2277 | 0.1413 | 0.001344 | 1.792 (1.25-2.569) |
|  | 17 | rs72851128^#^ | A | 0.09459 | 0.04646 | 0.004567 | 2.144 (1.252-3.672) |
|  | 17 | rs74662194 | A | 0.2037 | 0.1717 | 0.2657 | 1.234 (0.8519-1.786) |
|  | 17 | rs79652119 | A | 0.1802 | 0.1379 | 0.107 | 1.374 (0.9326-2.023) |
|  | 17 | rs12950256 | A | 0.1682 | 0.1002 | 0.004274 | 1.816 (1.2-2.748) |
|  | 17 | rs77256003 | A | 0.1697 | 0.1478 | 0.4135 | 1.179 (0.7943-1.75) |
|  | 17 | rs4479291 | A | 0.1682 | 0.1382 | 0.2565 | 1.261 (0.8442-1.884) |
|  | 17 | rs77848614 | A | 0.1321 | 0.1545 | 0.4064 | 0.8325 (0.5397-1.284) |
|  | 17 | rs7502642 | A | 0.25 | 0.2368 | 0.6757 | 1.074 (0.7676-1.504) |
|  | 17 | rs77221079^#^ | G | 0.04545 | 0.02551 | 0.1121 | 1.819 (0.8606-3.845) |
|  | 17 | rs72841253 | G | 0.09735 | 0.1212 | 0.3143 | 0.7821 (0.4841-1.264) |
|  | 17 | rs75092184 | A | 0.1396 | 0.09109 | 0.02935 | 1.619 (1.046-2.507) |
|  | 17 | rs2430508^#^ | G | 0.02655 | 0.04167 | 0.2888 | 0.6273 (0.263-1.496) |
|  | 17 | rs2429966 | A | 0.3227 | 0.3398 | 0.6283 | 0.9258 (0.6777-1.265) |
|  | 17 | rs116927798 | C | 0.1036 | 0.1318 | 0.253 | 0.761 (0.4759-1.217) |
|  | 17 | rs78802470^#^ | A | 0.09375 | 0.04361 | 0.002473 | 2.269 (1.318-3.906) |
|  | 17 | rs74852553^#^ | G | 0.01327 | 0.04388 | 0.03037 | 0.2931 (0.09013-0.9535) |
|  | 17 | rs116174686^#^ | G | 0.05 | 0.01524 | 0.001345 | 3.4 (1.54-7.508) |
|  | 17 | exm2272609 | C | 0.06757 | 0.08519 | 0.3871 | 0.7781 (0.4401-1.376) |
|  | 17 | rs78109261 | A | 0.1239 | 0.05769 | 0.000434 | 2.31 (1.433-3.724) |
|  | 17 | rs16976856^#^ | G | 0.02273 | 0.04786 | 0.09766 | 0.4626 (0.1818-1.177) |
|  | 17 | rs12451040 | C | 0.1216 | 0.1339 | 0.6257 | 0.8958 (0.5756-1.394) |
|  | 17 | rs2430532 | G | 0.06195 | 0.0568 | 0.7646 | 1.097 (0.5993-2.007) |
|  | 17 | rs2429938 | C | 0.05856 | 0.05466 | 0.8183 | 1.076 (0.5765-2.008) |
|  | 17 | rs2429931 | G | 0.05046 | 0.07389 | 0.2187 | 0.6661 (0.3472-1.278) |
|  | 17 | rs2430540 | C | 0.07273 | 0.06823 | 0.812 | 1.071 (0.6081-1.887) |
|  | 17 | rs2430542 | G | 0.07207 | 0.1065 | 0.1228 | 0.6517 (0.377-1.127) |
|  | 17 | rs2430543 | G | 0.0625 | 0.0596 | 0.8689 | 1.052 (0.5764-1.92) |
|  | 17 | rs2429953 | A | 0.2188 | 0.1752 | 0.1288 | 1.318 (0.9222-1.884) |
|  | 17 | rs117468022 | G | 0.04955 | 0.05364 | 0.8055 | 0.9197 (0.4723-1.791) |
|  | 17 | rs77163229 | A | 0.1036 | 0.05668 | 0.01055 | 1.924 (1.156-3.2) |
|  | 17 | rs12452827 | G | 0.05909 | 0.07215 | 0.4917 | 0.8076 (0.4387-1.487) |
|  | 17 | rs713122 | A | 0.2812 | 0.249 | 0.3172 | 1.18 (0.8528-1.634) |
|  | 17 | rs79163261 | A | 0.1606 | 0.164 | 0.9017 | 0.9752 (0.6544-1.453) |
|  | 17 | rs2529379 | A | 0.2965 | 0.2347 | 0.05204 | 1.374 (0.9964-1.895) |
|  | 17 | rs12453745 | G | 0.1045 | 0.127 | 0.3585 | 0.8023 (0.501-1.285) |
|  | 17 | rs4793377 | C | 0.1409 | 0.1626 | 0.4259 | 0.8447 (0.5573-1.28) |
|  | 17 | rs74904236 | A | 0.1355 | 0.1311 | 0.8626 | 1.039 (0.6738-1.602) |
|  | 17 | rs4793378 | A | 0.1773 | 0.1724 | 0.8644 | 1.034 (0.7045-1.518) |
|  | 17 | rs11871424 | G | 0.4732 | 0.4712 | 0.9564 | 1.008 (0.7536-1.349) |
|  | 17 | rs7213726 | G | 0.08108 | 0.1217 | 0.08596 | 0.6369 (0.3792-1.07) |
|  | 17 | rs76877588 | A | 0.08636 | 0.05894 | 0.1329 | 1.509 (0.8794-2.59) |
|  | 17 | rs16976977 | A | 0.3056 | 0.2495 | 0.08883 | 1.324 (0.9577-1.829) |
|  | 17 | rs35304994 | G | 0.3829 | 0.4041 | 0.5605 | 0.915 (0.6784-1.234) |
|  | 17 | rs12951391 | A | 0.2706 | 0.1951 | 0.01306 | 1.531 (1.092-2.146) |
|  | 17 | rs9905128 | A | 0.5409 | 0.4776 | 0.08968 | 1.289 (0.9611-1.728) |
|  | 17 | rs2158917 | A | 0.3208 | 0.2403 | 0.01478 | 1.493 (1.08-2.063) |
|  | 17 | rs28542368 | C | 0.2454 | 0.1884 | 0.0574 | 1.401 (0.9884-1.985) |
|  | 17 | rs2015816 | G | 0.1682 | 0.2718 | 0.001388 | 0.5417 (0.3704-0.7922) |
|  | 17 | rs7217932 | A | 0.482 | 0.502 | 0.5894 | 0.9229 (0.6895-1.235) |
|  | 17 | rs7209111 | G | 0.2589 | 0.2424 | 0.6031 | 1.092 (0.7831-1.523) |
|  | 17 | rs6501514 | A | 0.2207 | 0.2337 | 0.6794 | 0.9289 (0.6546-1.318) |
|  | 17 | rs2190492 | A | 0.5318 | 0.443 | 0.01684 | 1.428 (1.065-1.915) |
|  | 17 | rs28415098 | G | 0.03182 | 0.05306 | 0.1879 | 0.5865 (0.2627-1.309) |
|  | 17 | rs73996916 | C | 0.0991 | 0.09878 | 0.9884 | 1.004 (0.6163-1.634) |
|  | 17 | rs2160441 | A | 0.4144 | 0.3661 | 0.1791 | 1.226 (0.9107-1.65) |
|  | 17 | rs2193056 | G | 0.1204 | 0.1072 | 0.5757 | 1.139 (0.7211-1.801) |
|  | 17 | rs2216167 | G | 0.473 | 0.4572 | 0.6708 | 1.065 (0.7957-1.426) |
|  | 17 | rs12451606 | G | 0.2336 | 0.2211 | 0.6895 | 1.074 (0.7565-1.525) |
|  | 17 | rs9892959 | C | 0.2143 | 0.2114 | 0.9235 | 1.017 (0.7142-1.45) |
|  | 17 | rs7218051 | A | 0.5727 | 0.4683 | 0.005114 | 1.522 (1.133-2.044) |
|  | 17 | rs9916207 | A | 0.2812 | 0.2118 | 0.02466 | 1.456 (1.048-2.023) |
|  | 17 | rs34578823 | G | 0.08333 | 0.09714 | 0.5305 | 0.845 (0.4989-1.431) |
|  | 17 | rs2342302 | G | 0.3929 | 0.5213 | 0.0005192 | 0.5942 (0.442-0.7987) |
|  | 17 | rs9894442 | G | 0.06364 | 0.1184 | 0.01811 | 0.5059 (0.2848-0.8988) |
|  | 17 | rs7207922 | A | 0.08796 | 0.1087 | 0.3671 | 0.7905 (0.4738-1.319) |
|  | 17 | rs9911193 | G | 0.07207 | 0.1189 | 0.04421 | 0.5756 (0.334-0.9917) |
|  | 17 | rs16977050 | G | 0.06757 | 0.1116 | 0.05185 | 0.5771 (0.3295-1.011) |
|  | 17 | rs8077886 | G | 0.05357 | 0.06061 | 0.6873 | 0.8774 (0.4638-1.66) |
|  | 17 | rs9913936 | A | 0.3364 | 0.3469 | 0.7672 | 0.9544 (0.7008-1.3) |
|  | 17 | rs76299801^#^ | A | 0.08333 | 0.04192 | 0.01104 | 2.078 (1.169-3.692) |
|  | 17 | rs9907198 | A | 0.3063 | 0.3643 | 0.1028 | 0.7706 (0.5631-1.054) |
|  | 17 | rs9903394 | G | 0.2752 | 0.3927 | 0.001157 | 0.5872 (0.4249-0.8115) |
|  | 17 | rs74620814^#^ | A | 0.07273 | 0.01717 | 4.74E-06 | 4.489 (2.231-9.032) |
|  | 17 | rs9913711 | G | 0.367 | 0.387 | 0.5828 | 0.9184 (0.6777-1.245) |
|  | 17 | rs9906743 | A | 0.02358 | 0.05556 | 0.05239 | 0.4106 (0.1624-1.038) |
|  | 17 | rs9904984^#^ | G | 0.004545 | 0.04656 | 0.003564 | 0.09351 (0.01283-0.6817) |
|  | 17 | rs1042673 | G | 0.4037 | 0.3075 | 0.006117 | 1.524 (1.126-2.063) |

*This table doesnot contains the varaints that were found significantly associated with AIS in the Northwest Indian population. Significanlty associated varaints are enlisted in table1 of the mansuscript.

^#^ The variants that were not able to meet the filtering criteria during the analysis.
